# Incidental Microscopic Bile Duct Tumor Thrombi in Hepatocellular Carcinoma after Curative Hepatectomy: A Matched Study

**DOI:** 10.1097/MD.0000000000000450

**Published:** 2015-02-13

**Authors:** Jong M. Kim, Choon H. D. Kwon, Jae-Won Joh, Dong H. Sinn, Jae B. Park, Joon H. Lee, Sung J. Kim, Seung W. Paik, Cheol K. Park, Byung C. Yoo

**Affiliations:** From the Department of Surgery (JMK, CHDK, J-WJ, JBP, SJK); Department of Medicine, Division of Gastroenterology (DHS, JHL, SWP, BCY); and Department of Pathology, Samsung Medical Center, Sungkyunkwan University School of Medicine, Seoul, Korea (CKP).

## Abstract

In patients with hepatocellular carcinoma (HCC), the presence of bile duct tumor thrombi (BDTT) in the major bile ducts indicates poor prognosis compared with that of HCC patients without BDTT. However, the prognostic significance of incidental microscopic BDTT in the peripheral bile ducts after curative liver resection is not known. We compared the outcomes of HCC patients with and without microscopic BDTT in the peripheral bile ducts who underwent hepatectomy.

The electronic medical records of 31 patients with microscopic BDTT (BDTT group) were retrospectively reviewed. To compare the surgical outcomes, 62 patients (No BDTT group) were randomly chosen from the remaining HCC patients without BDTT based on age, sex, etiology of HCC, tumor size, tumor number, and modified Union for International Cancer Control T staging.

The 1-year, 2-year, and 3-year disease-free survival rates and overall survival rates were 54.8%, 34.0%, 34.0% and 90.1%, 69.2%, 61.0% in the BDTT group and 66.8%, 59.2%, 42.3% and 86.4%, 84.4%, 84.4% in the No BDTT group (*P* = 0.089 and *P* = 0.014, respectively). The overall survival curve in the No BDTT group was higher than that in the BDTT group. Multivariate analysis revealed that predisposing factors for tumor recurrence after curative liver resection included increased levels of the protein induced by vitamin K antagonist-II (PIVKA-II), tumor grades 3 and 4, and the presence of BDTT.

This study demonstrates that HCC prognosis is worse in patients with incidental microscopic BDTT in the peripheral bile ducts than it is in those without BDTT. The presence of BDTT should therefore be considered when evaluating a patient's HCC prognosis after curative hepatectomy.

## INTRODUCTION

Portal vein tumor thrombi are poor prognostic factors in patients with hepatocellular carcinoma (HCC).^1^ However, bile duct tumor thrombi (BDTT) due to HCC are rare and poorly characterized. Patients with BDTT in the major bile ducts often present with jaundice and are sometimes misdiagnosed as having cholangiocarcinoma or choledocholithiasis. When a tumor thrombus involves a major bile duct in HCC, patients have a poorer prognosis than they do when they have HCC without BDTT.^2,3^

BDTT can be the result of a fragment of necrotic tumor that breaks off of a proximal intraductal growth, or a tumor hemorrhage that partially or completely fills the biliary tract with blood clots.^4^

Most prior literature addresses HCC with BDTT in the major biliary branches, which produces obstructive jaundice.^2–11^ However, incidentally discovered microscopic BDTT in peripheral bile ducts after curative liver resection has not been previously studied. There is little information regarding the prognosis after HCC resection with microscopic BDTT.

In this study, we retrospectively reviewed the clinical and pathological characteristics of HCC patients with microscopic BDTT in peripheral bile ducts who underwent surgical treatment at our hospital. The outcomes of these patients were compared with those of patients without microscopic BDTT who were matched for age, sex, HCC etiology, tumor size, tumor number, and modified Union for International Cancer Control (UICC) T staging.

## MATERIALS AND METHODS

### Patients

A total of 1222 patients who underwent surgical resection of HCC between January 2005 and December 2010 were included. Exclusion criteria included: pathology revealing mixed HCC and cholangiocarcinoma; younger than 18 years; previous locoregional therapy such as hepatectomy, radiation, transarterial chemoembolization (TACE), radiofrequency ablation (RFA), percutaneous ethanol injection, or intraoperative RFA; preoperative obstructive jaundice caused by bile duct occlusion from macroscopic bile duct tumor thrombus; or loss to follow-up after hepatectomy. The demographic characteristics, preoperative laboratory data, and pathologic data from 31 patients with microscopic BDTT were collected from the electronic medical record (EMR). To compare the surgical outcomes, 62 patients were randomly chosen from the remaining HCC patients without BDTT and were matched 2:1 with patients with BDTT by age, sex, HCC etiology, tumor size, tumor number, and modified UICC T staging. None of the patients in either group received postoperative adjuvant therapy prior to recurrence. Our study was approved by the institutional review board of Samsung Medical Center in Seoul (SMC 2014-07-132).

### Surgery and Pathology

Liver function was evaluated using the Child-Pugh classification system. Patients were considered for tumor resection if they had a single mass in one liver lobe that was visualized on preoperative imaging without evidence of extrahepatic or nodal disease. Patients were required to have Child-Pugh class A liver function, although selected patients with Child-Pugh class B liver function were also included. Patients were not considered for resection if they had a serum total bilirubin level ≥1.5 mg/dL, indocyanin green (ICG) scores ≥20%, or ascites. Patients with gross vascular invasion on imaging were only considered for resection if the main portal vein and the portal branch to the remaining liver lobe were patent. Other parameters that were recorded include the platelet count, serum albumin level, total bilirubin level, aspartate transaminase, alanine transaminase, alkaline phosphatase, creatinine, international normalization ratio, alpha-fetoprotein (AFP) level, and protein induced by vitamin K antagonist-II (PIVKA-II) level.

Standard operative techniques for hepatectomy were used. Adequate mobilization was achieved based on the part of liver to be resected. When possible, the portal vein and hepatic vein were selectively clamped. If this clamping was not feasible, the intermittent Pringle maneuver was performed. A Cavitron Ultrasonic Surgical Aspirator (CUSA) was used to perform parenchymal transection under low central venous pressure. Tumorous livers were carefully resected to minimize tumor spillage into the peripheral duct. Major hepatectomy was defined as the resection of ≥3 Couinaud segments and minor hepatectomy was defined as the resection of <3 segments. Anatomic resections involved resection of the tumor with its related portal vein branches and corresponding hepatic territory. Both peripheral tumors and central tumors were treated with nonanatomic resections. Peripheral tumors and tumors with extrahepatic growth were treated by partial hepatectomy because this method achieved sufficient surgical outcomes. Central tumors near the hepatic hilum or major vessels were all treated by enucleation. In these cases, only removing enough of the liver to obtain adequate margins was too difficult or too dangerous.

Postoperatively histological assessment and reporting included the evidence of fibrosis, lobular activity, tumor diameter, number of tumors, encapsulation, portal vein invasion, BDTT, microvascular invasion, intrahepatic metastasis, multicentric occurrence, and serosal tumor involvement. In our study, BDTT was defined when it was present in the peripheral ducts beyond the secondary duct division, regardless of bile duct lumen obstruction. Intrahepatic metastasis and multicentric occurrence were defined based on guidelines from the Liver Cancer Study Group of Japan.^12^

The Edmonson-Steiner was used to grade the HCC^13^ as well differentiated (grade I), moderately differentiated (grade II), or poorly differentiated (grade III, IV). Modified UICC staging was used to stage the HCC.^14^ The Ludwig-Batts scoring system was used to assess and grade the hepatic fibrosis (stage) on a scale of 0–4 with F0 = absent, F1 = portal fibrosis, F2 = periportal fibrosis, F3 = bridging fibrosis, and F4 = cirrhosis. Tumor recurrence and survival data were also recorded.

### Surveillance After Surgical Resection

Patients were followed postoperatively every 2 to 3 months after surgery. Follow-up evaluation included physical examination, AFP, PIVKA-II, liver function tests, and chest X-rays. Abdominal computed tomography was performed every 3 months or when recurrence was suspected. If the computed tomography did not show definitive evidence of recurrence, magnetic resonance imaging and/or positron emission tomography scans were performed. Detailed information on patients found to have a recurrence was recorded. Patients with intrahepatic recurrences were treated with RFA, TACE, or sorafenib according to their functional liver reserve and recurrence pattern. Follow-up time was defined as the time from surgery to the time of the last follow-up or death. No patients were lost to follow-up and all 93 patients were included in the survival analysis.

### Statistical Analysis

Patient data were collected retrospectively from the electronic medical records (EMRs). Patients were matched based on multivariate logistic regression using age, sex, HCC etiology, tumor size, tumor number, and modified UICC T staging. We performed 1:2 fixed ratio nearest neighbor matching between HCC patients with and without microscopic BDTT. Categorical variables were expressed as percentages and were compared using the *χ*^2^ test or Fisher exact test. Continuous variables were expressed as medians and ranges. They were compared using the Mann-Whitney *U* test. Patient survival and recurrence were calculated using the Kaplan-Meier method and were compared using the log-rank test. Clinical and pathological variables found to have prognostic significance in univariate analysis were entered into a Cox multivariate proportional hazards model to determine factors that independently predicted HCC recurrence. Statistical significance was defined by *P* values *<*0.05. Data analysis was performed using SPSS 20.0 (SPSS, Chicago, IL, USA).

## RESULTS

All patients had a single tumor and were Child-Pugh class A. None of the patients had preoperative obstructive jaundice. A comparison of the baseline HCC features in patients with (BDTT group) and without microscopic BDTT (No BDTT group) is shown in Table 1. There were no significant differences between the 2 groups with regard to sex, age, HCC etiology, AFP or PIVKA-II levels, or preoperative laboratory findings except for serum albumin and gamma-glutamyl transpeptidase (GGT). The serum albumin levels in the BDTT group were lower than those in the No BDTT group (3.9 vs 4.2, *P* = 0.040). In contrast, the serum GGT levels in the BDTT group were higher than those in the No BDTT group (163 vs 86, *P* = 0.016).

**TABLE 1:**
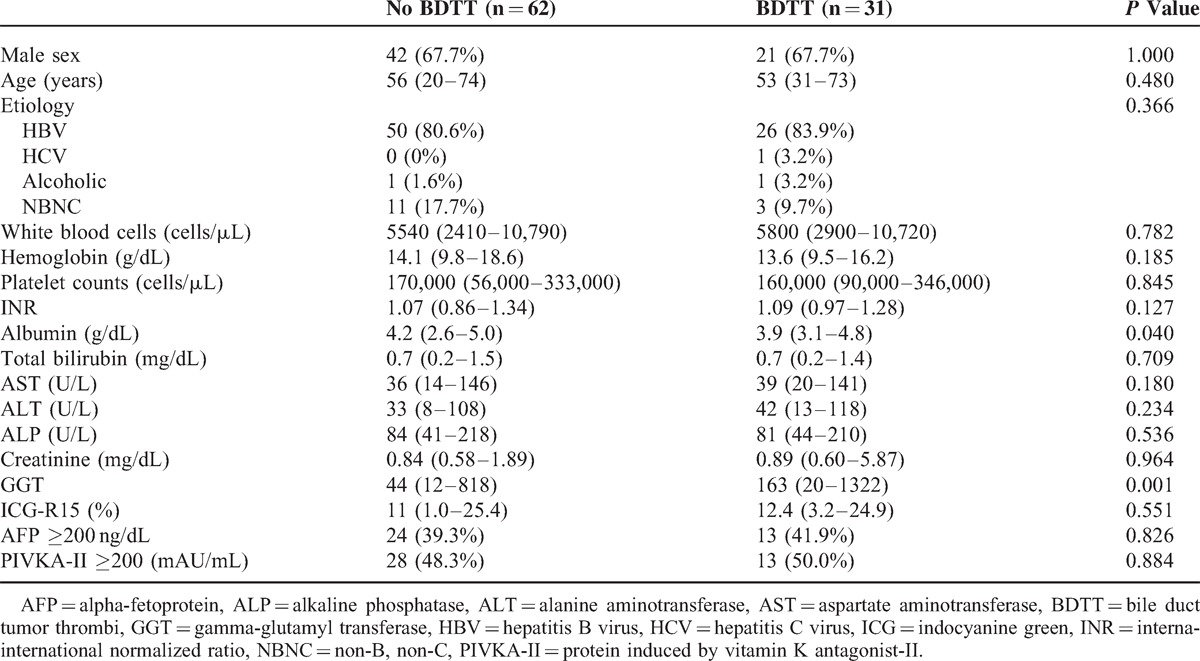
Baseline Patient Characteristics (Median and Range)

**FIGURE 1:**
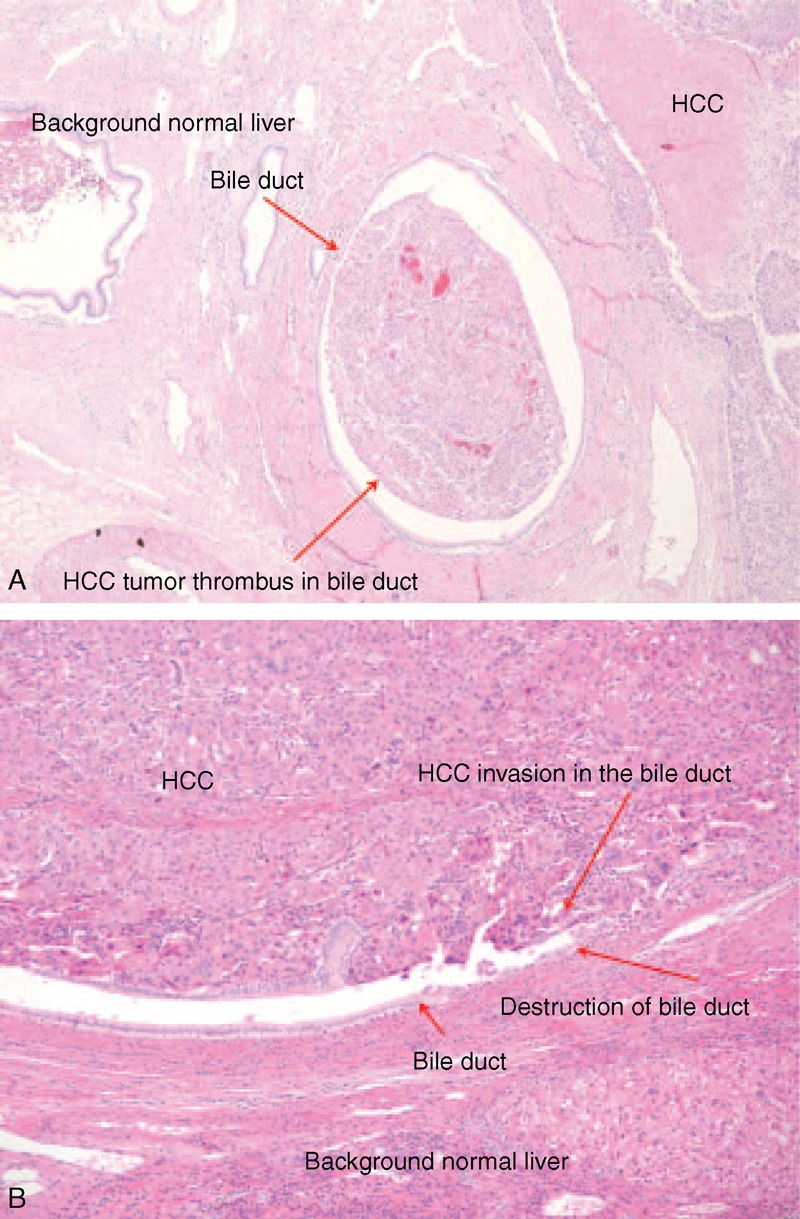
HCC bile duct thrombus in the bile duct lumen. (A) Bile duct mucosa was intact (hematoxylin-eosin staining, original magnification 40×). (B) HCC invasion and destruction of the bile duct wall and tumor thrombi in the bile duct lumen (hematoxylin-eosin staining, original magnification 100×). HCC = hepatocellular carcinoma

The perioperative and pathologic features of HCC patients with or without BDTT are shown in Table 2 and Figure 1. Tumor size did not differ significantly between the No BDTT and the BDTT groups (5.0 cm vs 4.8 cm, *P* = 0.912). The procedure performed, including either major liver resection, anatomical liver resection, or resection-free margins, did not differ between the 2 groups. There were no significant differences in tumor grade, encapsulation, microvascular invasion, portal vein invasion, or intrahepatic metastasis between the 2 groups. No multicentric occurrence or serosal tumor involvement was observed. The fibrosis, lobular activity, and periportal activity in the normal liver also did not differ from that of the resected specimens from both groups. In the BDTT group, there was no grossly close contact between the thrombi and the bile duct wall. In addition, there was no microscopic bile duct infiltration on any of the tumor slides.

**TABLE 2:**
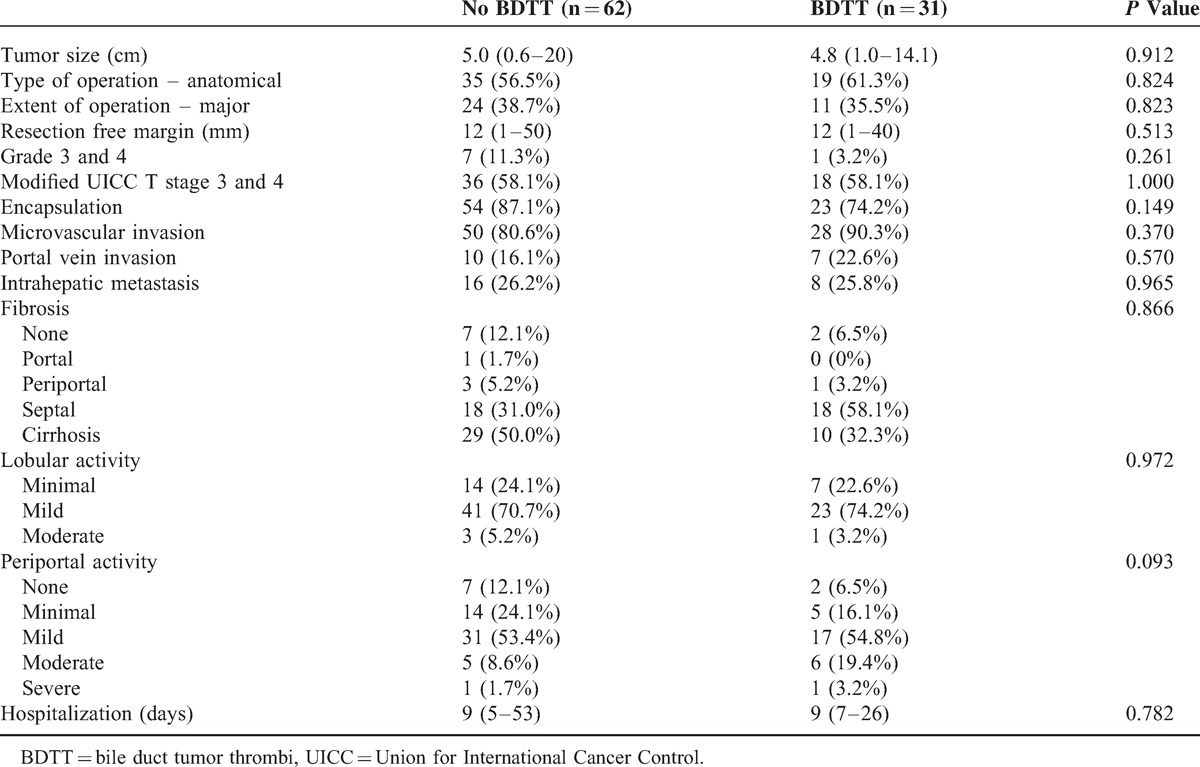
Perioperative Characteristics (Median and Range)

**FIGURE 2:**
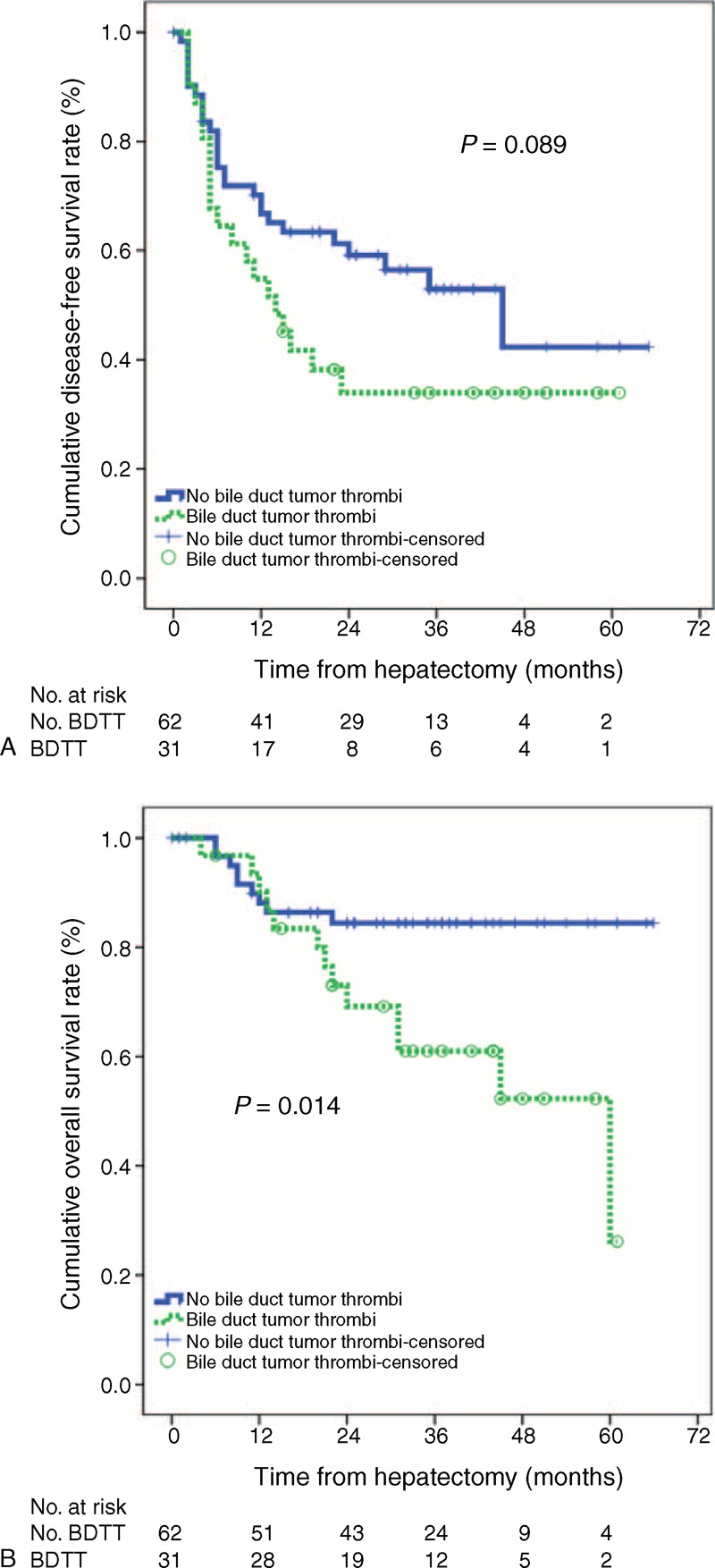
(A) Disease-free survival and (B) overall survival.

Study participants were not matched by their resection date. The median follow-up durations were 30 and 31 months for the No BDTT and BDTT groups, respectively (*P* = 0.754). In the BDTT group, disease-free survival rates were 54.8% at 1 year, and 34.0% at 2 and 3 years and overall survival rates were 90.1% at 1 year, 69.2% at 2 years, and 61.0% at 3 years. For the No BDTT group, disease-free survival rates were 66.8% at 1 year, 59.2% at 2 years, and 42.3% at 3 years and overall survival rates were 86.4% at 1 year and 84.4% at 2 and 3 years (Figure 2). Thus, disease-free survival and overall survival in the No BDTT group were higher than those in the BDTT group, although the difference in disease-free survival was not significant (*P* = 0.089). The overall survival difference between the 2 groups was significant (*P* = 0.014). The intrahepatic, systemic, and concurrently intrahepatic and systemic recurrences were not different between the 2 groups (Table 3).

**TABLE 3:**
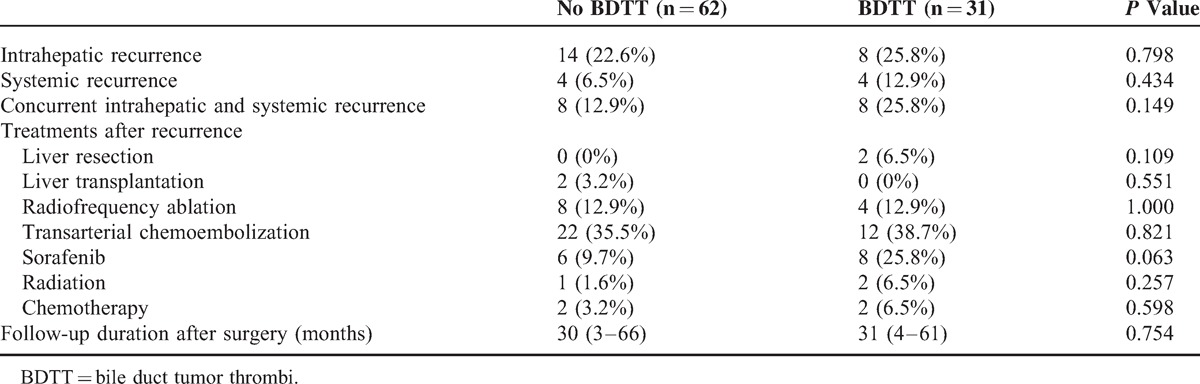
Tumor Recurrence After Liver Resection (Median and Range)

We analyzed the known risk factors for tumor recurrence. Multivariate analysis showed that the predisposing factors for tumor recurrence after curative liver resection included increased PIVKA-II levels (odds ratio [OR] 3.223; 95% confidence interval [CI] 1.155–8.993; *P* = 0.042), grade 3 and 4 (OR 4.219; 95% CI 1.050–16.942; *P* = 0.025), and the presence of BDTT (OR 3.135; 95% CI 1.136–8.649; *P* = 0.027) (Table 4).

**TABLE 4:**
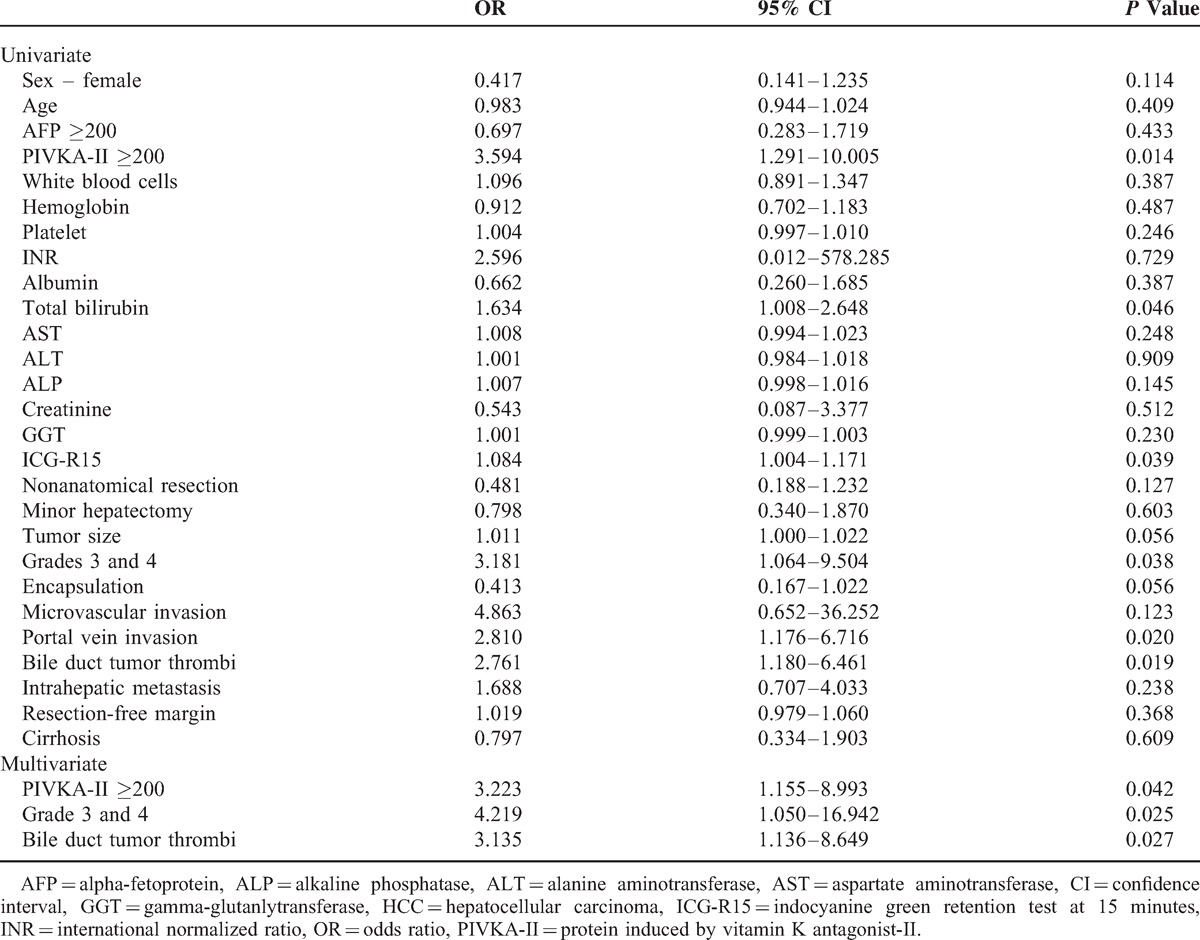
Risk Factors for HCC Recurrence in Patients After Liver Resection

## DISCUSSION

Most previous studies have only addressed tumor invasion into large bile ducts, such as the common bile duct up to the first branches of the hepatic duct.^5,6,8^ Peripheral bile duct invasion, in contrast, has not been well studied. One study reported that macroscopic bile duct invasion was associated with a favorable prognosis in patients treated with extensive and curative surgical resection; these patients also had satisfactory hepatic function reserve without intrahepatic metastases.^15^ Most studies have shown that HCC with macroscopic BDTT is characteristically aggressive and has a poor prognosis.^2–4,10^ A recent study showed that most tissues in HCC tumors with macroscopic BDTT expressed high levels of liver stem cell markers.^4^

Despite recent improvements in imaging techniques, it is difficult to detecting BDTT in the peripheral bile ducts in HCC. We evaluated HCC patients with peripheral microscopic BDTT. The clinical prognostic factors that are generally accepted as unfavorable including the presence of microscopic vascular invasion or portal vein invasion, tumor capsule absence, tumor grade, AFP, and PIVKA-II were not significantly different between the BDTT and No BDTT groups. We found that peripheral microscopic BDTT has a negative impact on the prognosis of HCC patients. Subgroup analysis for modified UICC T1 and T2 staging and tumor size <5 cm showed that peripheral microscopic BDTT was associated with tumor recurrence after curative liver resection.

There is inconsistent evidence regarding resection of and survival after HCC with or without associated BDTT. The treatment strategies for HCC were sequential. Unfortunately, patient survival after liver resection in HCC patients could not be measured because recurrent HCC patients underwent multiple treatment strategies. Some studies reported that there is no significant survival difference between patients with and without BDTT after hepatic resection. This finding suggests that bile duct thrombi might be of little importance in terms of prognosis.^5,6^ In contrast, other studies have reported that HCC patients with biliary tumor thrombi after surgery have significantly lower overall survival than do similar patients without thrombi (5-year survival, 6.7% with thrombi vs 33.0% without).^2,10^ In our study, the 1-year, 2-year, and 3-year disease-free survival and overall survival rates of HCC patients with microscopic BDTT were 54.8%, 34.0%, 34.0% and 90.1%, 69.2%, 61.0%, respectively. The corresponding 1-year, 2-year, and 3-year disease-free survival and overall survival rates of matched HCC patients without microscopic BDTT were 66.8%, 59.2%, 42.3% and 86.4%, 84.4%, 84.4%, respectively. These results indicated that the HCC patients with microscopic BDTT had poorer prognosis than patients without microscopic BDTT.

Another study reported that there was no microscopic infiltration of tumor cells into the bile duct wall found.^5^ In addition, most BDTT in the large bile ducts is easy to remove because the thrombi do not usually adhere tightly to bile duct wall.^11^ Most cases of HCC with microscopic BDTT in the present study were not infiltrative. However, in few cases, there was infiltration and destruction of the peripheral bile ducts. We found that the disease-free survival and overall survival in the No BDTT group were higher than those in the BDTT group. In addition, multivariate analysis showed that microscopic BDTT was associated with tumor recurrence after surgery.

Several studies have reported clinical and pathologic differences between HCC with and without BDTT to those patients without HCC altogether.^2,3,7,9^ Some studies found that all cases of HCC with BDTT lacked capsular formation.^7,9^ The proportion of encapsulation in our study was 74.2% because we included cases with microscopic BDTT. BDTT can occur when the primary tumor is very small.^16^ However, the mechanism of BDTT formation is unclear and its clinical and pathological characteristics remain to be defined. Portal vein invasion has been previously identified as an independent marker of poor prognosis in HCC with BDTT.^3^ However, we found that portal vein invasion was not associated with HCC recurrence after curative hepatic resection. In addition, HCC with tumor thrombi is generally associated with poor prognosis, most likely because it reflects early intrahepatic recurrence.^17^ However, we found that the incidence of intrahepatic recurrence was not significantly different in HCC patients with and without microscopic BDTT. HCC patients with microscopic BDTT had a high incidence of tumor recurrence after curative liver resection. Therefore, HCC patients with BDTT should have frequent follow-up after surgery to detect recurrence as early as possible.

In the Japanese TNM staging system, the presence of BDTT increases the T classification by 1 grade.^12^ In contrast, the American Joint Committee on Cancer/International Union Against Cancer does not even consider bile duct invasion when assigning stage with its TNM staging system.^16^ Our results suggest that microscopic BDTT in the small peripheral ducts should be considered during staging, just as biliary tumor thrombi in the large branches are considered.

Our study demonstrates that HCC patients with microscopic BDTT in the peripheral bile ducts had a higher incidence of HCC recurrence than did patients with HCC without BDTT. HCC patients were matched for sex, age, HCC etiology, tumor size, tumor number, and modified UICC T staging. Given its effect on prognosis, patients with incidentally discovered microscopic bile duct tumor thrombi in the peripheral bile ducts should be carefully monitored for recurrence. However, this study included a small number of cases from a single center; therefore, larger multicenter clinical studies are needed to define the clinical and pathological characteristics of HCC with BDTT.
